# A SARS-CoV-2: Companion Animal Transmission and Variants Classification

**DOI:** 10.3390/pathogens12060775

**Published:** 2023-05-29

**Authors:** Rachana Pandit, Qiana L. Matthews

**Affiliations:** 1 Microbiology Program, Department of Biological Sciences, College of Science, Technology, Engineering and Mathematics, Alabama State University, Montgomery, AL 36104, USA; 2 Department of Biological Sciences, College of Science, Technology, Engineering and Mathematics, Alabama State University, Montgomery, AL 36104, USA

**Keywords:** human coronavirus, SARS-CoV, MERS-CoV, SARS-CoV-2, pathogenesis, COVID-19, animal host, variants of SARS-CoV-2

## Abstract

The continuous emergence of novel viruses and their diseases are a threat to global public health as there have been three outbreaks of coronaviruses that are highly pathogenic to humans in the span of the last two decades, severe acute respiratory syndrome (SARS)-CoV in 2002, Middle East respiratory syndrome (MERS)-CoV in 2012, and novel SARS-CoV-2 which emerged in 2019. The unprecedented spread of SARS-CoV-2 worldwide has given rise to multiple SARS-CoV-2 variants that have either altered transmissibility, infectivity, or immune escaping ability, causing diseases in a broad range of animals including human and non-human hosts such as companion, farm, zoo, or wild animals. In this review, we have discussed the recent SARS-CoV-2 outbreak, potential animal reservoirs, and natural infections in companion and farm animals, with a particular focus on SARS-CoV-2 variants. The expeditious development of COVID-19 vaccines and the advancements in antiviral therapeutics have contained the COVID-19 pandemic to some extent; however, extensive research and surveillance concerning viral epidemiology, animal transmission, variants, or seroprevalence in diverse hosts are essential for the future eradication of COVID-19.

## 1. Introduction to the Human Coronavirus

Coronaviruses (CoVs) are the largest family of enveloped, single-stranded (ss), positive-sense RNA viruses causing respiratory, gastrointestinal, neurological, and other systematic diseases in a broad range of human and non-human hosts such as birds, companion, farm, zoo, or wild animals. The term “coronavirus” was given for the “crown”-shaped spiked complexes projected from the outer surface of the virion when observed under an electron microscope, and these spikes are attributed to the large type 1 transmembrane spike (S) glycoprotein that is responsible for viral attachment and host entry and subsequent infections. CoVs belong to the *Orthocoronavirinae* subfamily within the *Coronaviridae* family in the *Nidovirales* order. The *Orthocoronavirinae* subfamily is further classified into four genera: *Alphacoronavirus*, *Betacoronavirus*, *Gammacoronavirus,* and *Deltacoronavirus*, according to the International Committee on the Taxonomy of Viruses (ICTV) [1,2]. Alpha and beta CoVs mostly infect mammals such as bats, rodents, civets, cattle, horses, pigs, and humans, while gamma and delta CoVs mostly cause diseases in birds and some mammals such as pigs [3,4]. To date, seven human coronaviruses (HCoVs) are documented in the literature that have crossed the species barrier to infect humans, resulting in severe disease. Of them, human coronavirus (HCoV)-229E and HCoV-NL63 are alpha CoVs while HCoV-OC43, HCoV-HKU1, severe acute respiratory syndrome (SARS)-CoV, Middle East respiratory syndrome (MERS)-CoV, and novel SARS-CoV-2 belongs to the beta CoVs genera [5]. The latter three CoVs are major CoVs that have appeared in the 21st century and have caused severe diseases affecting public health worldwide during their outbreaks.

## 2. Coronavirus Disease-2019 (COVID-19)

COVID-19, a severe respiratory disease caused by a novel SARS-CoV-2 infection, has been causing the ongoing pandemic since its origin in Wuhan, Hubei Province in China, in December 2019. The genomic and phylogenetic studies have revealed that the outbreak was caused by a novel CoV, named 2019-nCoV initially and now globally known as SARS-CoV-2 announced by the World Health Organization (WHO) [1,6,7]. SARS-CoV-2 is the third major human CoV outbreak preceding SARS-CoV in 2002 and MERS-CoV in 2012 in the 21st century. The WHO declared the illness caused by novel SARS-CoV-2 as Coronavirus Disease 2019 and its pandemic a Public Health Emergency of International Concern in January 2020 [8,9]. As of the 17th of May 2023, a total of 766,440,796 confirmed COVID cases, including 6,932,591 deaths (~1% fatality rate), have been reported worldwide by the WHO [10]. As the world has been amid a COVID-19 pandemic since 2020, many organizations have come forward together to develop and deploy effective vaccines and antiviral therapeutics. The WHO has listed 382 total COVID-19 candidate vaccines, with 183 in the clinical phase and 199 in the pre-clinical phase as of 30 March 2023 [11]. COVID-19 vaccines such as the Pfizer-BioNTech vaccine (emergency use approval (EUA) by 31 December 2020), Oxford-AstraZeneca/AZD1222 vaccine (EUA by 16 February 2021), Johnson & Johnson’s Janssen/AD26.COV.S vaccine (EUA by 12 March 2021), Moderna’s mRNA1273 vaccine (EUA by 30 April 2021), Sinopharma COVID-19 vaccine (EUA by 7 May 2021), Sinovac-CoronaVac vaccine (EUA by 1 June 202), Bharat Biotech’s BBV152 COVAXIN vaccine (EUA by 3 November 2021), Covovax (NVX-CoV2373) vaccine (EUA by 17 December 2021) and Nuvaxovid (NVX-CoV2373) vaccine (EUA by 20 December 2021) have been validated for use by the WHO (given EUA or emergency use listing) against COVID-19 globally [11,12]. As of the 9th of May 2023, a total of 13,350,530,518 vaccine doses have been administered worldwide, reported by the WHO [12]. Furthermore, the booster immunization for COVID-19 vaccines has been approved to restore protection against COVID-19 which might have waned over time since the primary series vaccination. As of mid-May 2023, around 70% of the world population has received at least one dose of a COVID-19 vaccine in 203 countries. About 65% are fully vaccinated, and 33% of the global population has been administered an additional booster dose [13].

## 3. Morphology, Genome Organization, and Pathogenesis of SARS-CoV-2

**Figure 1:**
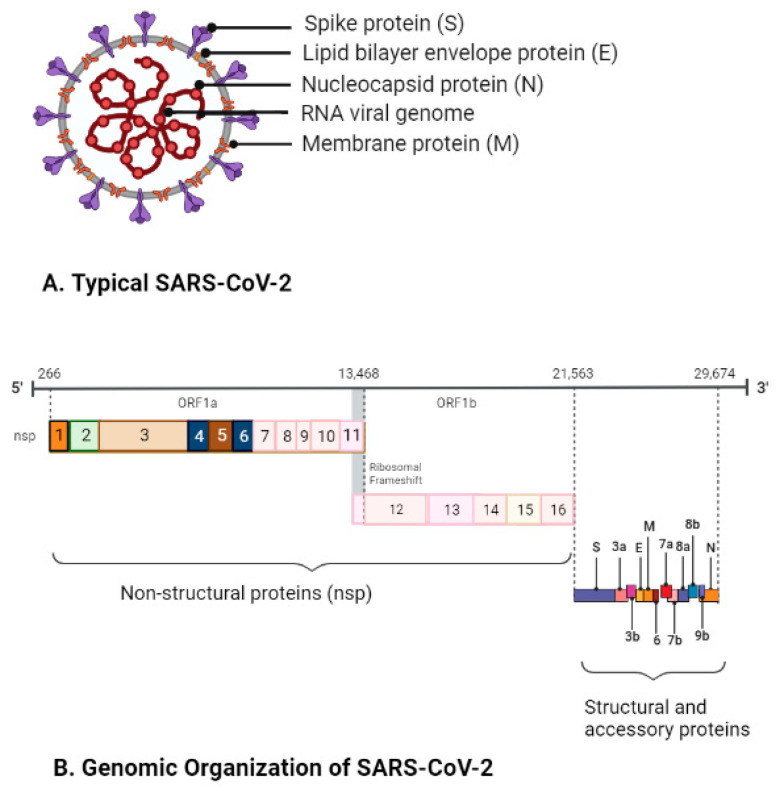
**Overview of SARS-CoV-2.** (**A**) Schematic of a typical SARS-CoV-2. SARS-CoV-2 is an enveloped, spherical-shaped, positive-sense RNA virus. (**B**) Genomic organization of the SARS-CoV-2. The SARS-CoV-2 genome is approximately 30 kB long and is comprised of a 5′-UTR, 6–15 ORFs, and ends with a 3′-UTR. The ORFs encode four structural proteins (S, Envelop E, Membrane M, and Nucleocapsid N), 16 non-structural proteins (nsps), and many accessory proteins (figure created with Biorender.com and accessed on 11 May 2023).

SARS-CoV-2 is an enveloped, spherical-shaped, positive-sense RNA virus of approximately 30 kB genome size and belongs to the *Betacoronavirus* genera of the *Sarbecovirus* subgenus of the *Coronaviridae* family in the *Nidovirales* order (Figure 1A,B) [7,13]. The structure and genomic organization of SARS-CoV-2 are found to be similar to SARS-like-CoVs. For instance, SARS-CoV-2 has 89–96% sequence identity to bat SARS-like-CoV, 80% sequence similarity to human SARS-CoV, and 50% to MERS-CoV [14,15,16]. The SARS-CoV-2 genome is comprised of a 5′- untranslated region (UTR), 6–15 open reading frames, and ends with a 3′-UTR (Figure 1B). The ORFs encode structural proteins, non-structural proteins, and accessory proteins. While the four structural proteins, S, E, M, and N, collectively play a role in genomic stability, viral morphogenesis, and assembly, the sixteen nsps (nsps 1–16) are involved in SARS-CoV-2 genome replication, transcription, pathogenesis, and immunomodulation in the host. Likewise, accessory proteins are mostly involved in immunomodulation and host immune escape during viral pathogenesis and infection [17,18,19]. The functions of structural, non-structural, and accessory proteins of SARS-CoV-2 are summarized in Table 1.

**Figure 2:**
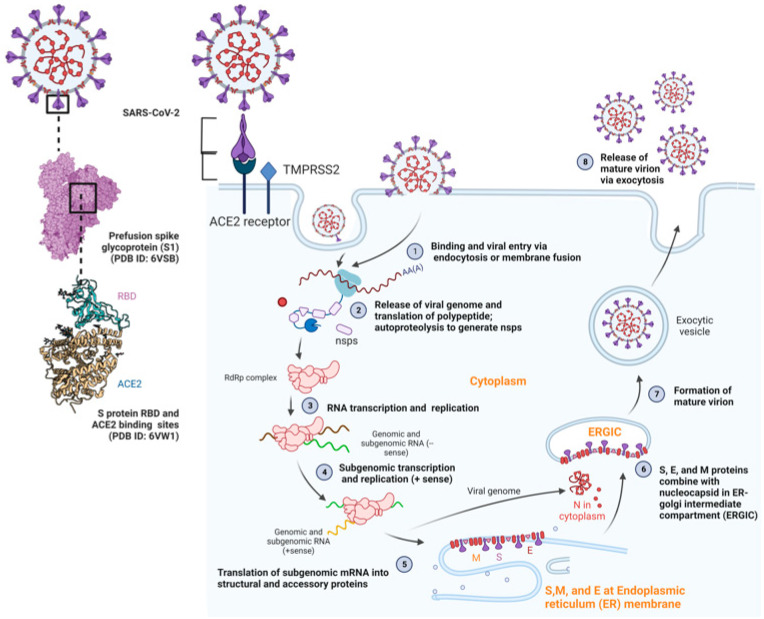
**Life cycle of SARS-CoV-2.** During the SARS-CoV-2 lifecycle, virus entry into the host can occur either via the plasma membrane route (early endocytic) or late endosomal entry route. In the plasma membrane route (for wild-type SARS-CoV-2 or previous SARS-CoV-2 variants before Omicron), the receptor binding domain on the S1 subunit of S protein of SARS-CoV-2 fuses with the host ACE2 (angiotensin-converting enzyme 2) receptor after activation of S protein by a host protease, type II transmembrane serine protease (TMPRSS2), and furin, co-expressed with ACE2 allowing the host cell entry via irreversible conformational changes in the S protein and early endocytosis (step 1). In the late endosomal entry route (for the Omicron variant), a virus binds to the ACE2 receptor, and the virus–ACE2 complex is internalized into the endosome through the endocytosis pathway. S protein is cleaved by cathepsin L and the fusion of viral and endosomal membranes forms a pore to release the viral genome (step 2). Ultimately, the viral ssRNA is then released in the cytoplasm, and translation and proteolysis of polypeptides occur to generate non-structural proteins and functional replicase (RdRp) (step 2). The replicase leads to viral RNA synthesis (steps 3 and 4). Translation of viral structural proteins (S, E, and M) takes place, and they are inserted and moved into the endoplasmic reticulum (ER) and the ER-Golgi intermediate compartment (ERGIC), respectively (step 5). The N proteins package genomic RNA and combines with other structural and accessory proteins in the ERGIC to assemble the virion (step 6). The mature virus is budded from the ERGIC membranes and released out of the cell via exocytosis (steps 7 and 8) (crystal structures adapted with permission from Lan et al. 2020, published by *Nature* 2020, Lan et al. [23] and figure created with Biorender.com and accessed on 8 May 2023).

The S protein of SARS-CoV-2 is a 1273 amino acid (aa) long transmembrane spike glycoprotein and is the most imperative protein of the virion. The S enables the binding of the virus to the host ACE2 for entering the host during virus infection and pathogenesis and the infection (Figure 2) [7,18,23]. It has two subunits, S1 (14–685 aa) and S2 (686–1273 aa), and the receptor binding domain (RBD) of the S1 subunit attaches to the host ACE2 receptor while the S2 subunit is involved during fusion [22,24]. The host receptor, ACE2 is a type 1 integral transmembrane carboxypeptidase protein expressed abundantly on the extracellular surface of human bronchial epithelial cells, lower bronchi type II pneumocytes, activated leukocytes and endothelial cells of the lower respiratory tract, including lungs, kidneys, alveoli, small intestine, liver, and renal tubes. ACE2 is primarily involved in the regulation of blood pressure and inflammatory response in normal body conditions [21,25]. The entry of SARS-CoV-2 can occur via furin S1-S2 junction cleavage and either type II transmembrane serine protease (TMPRSS2)-mediated S protein activation at the plasma membrane or via cathepsin L-mediated S protein activation through the endosomal pathway, without priming of TMPRSS2, at the target cell following ACE2 binding. The TMPRSS2 or cathepsin L-mediated cleavage is crucial for SARS-CoV-2 entry into host cells. While TMPRSS2 is present on the plasma membrane and mediates the plasma membrane entry route, cathepsin L in the endosomes follows the endosomal entry route [18,26]. Finally, the viral genome is released into the host cytoplasm for uncoating and replication. The viral proteins and genome synthesized at the replication site are then translocated to the ERGIC, in which virus assembly and budding occur. Matured virions are then released from the host cell via exocytosis (Figure 2) [27,28,29]. Given the sequence similarity and same mode of cellular entry, collective studies have reported that SARS-CoV-2 has similar pathogenesis as SARS-CoV, which attacks the respiratory track and potentially leads to severe respiratory complications, especially in severe cases [24,30].

## 4. Potential Animal Reservoirs of SARS-CoV-2

**Figure 3:**
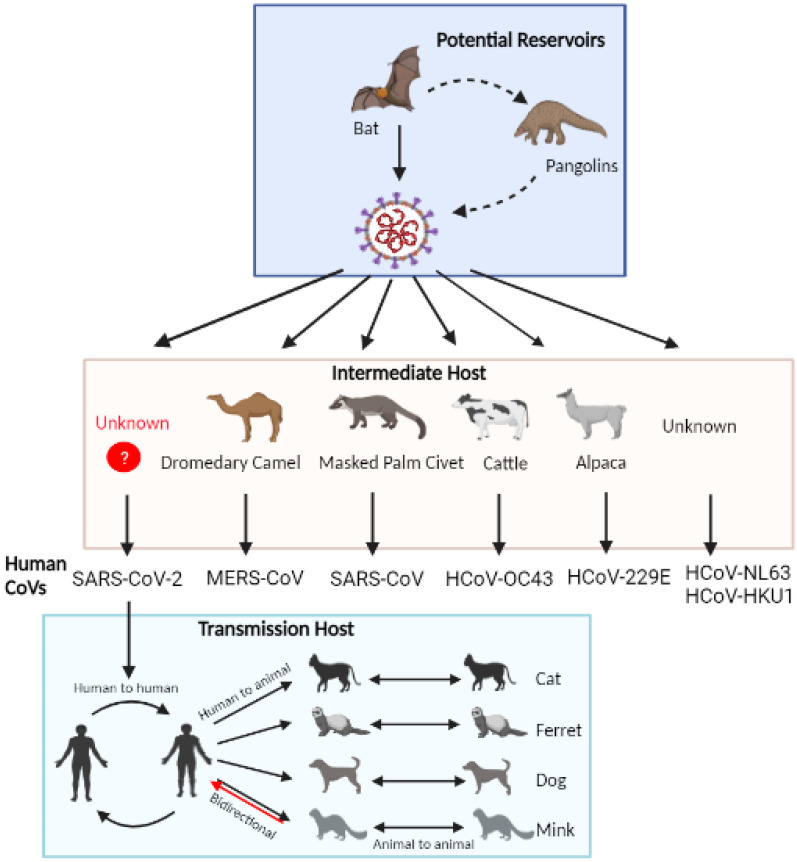
**Potential reservoirs, transmission hosts, and natural infections of SARS-CoV-2 to animals.** SARS-CoV-2 is believed to be zoonotic in origin and has spillover reports of SARS-CoV-2 circulating in a wide range of animal hosts, including humans, farm, and companion animals (figure created with Biorender.com and accessed on 10 May 2023).

As the world is amid a COVID-19 pandemic, the exact source of the SARS-CoV-2 origin and its intermediate host for crossing the species barrier to infect humans is still unknown. SARS-CoV-2 is most likely zoonotic in origin, and infections have been documented in human and non-human hosts such as companion, farm, zoo, or wild animals, indicative of its expanded host range compared to SARS-CoV and MERS-CoV. As of the 31 of December 2022, 36 countries have reported a total of 699 outbreaks in 26 different animals species, such as cats, dogs, minks, pet ferrets, otters, tigers, white-tailed deer, lynx, hamsters, monkeys, snow leopards, gorillas, and others, indicative of gradual host range expansion [31]. Correspondingly, it is imperative to review the potential animal reservoirs and naturally susceptible infections to companion animals and farm animals that are in prolonged contact with humans as probable reservoirs or hosts that can further lead to the emergence of new variants with spillback capacity to humans (Figure 3).

### 4.1. Bats

Initially, bats were presumed to be the primary reservoir of SARS-CoV-2 as well as SARS-CoV and MERS-CoV [14,32]. In the literature, there are several articles that implicated bats as being a natural reservoir host of many viruses, including CoVs. For instance, bats are the primary reservoirs for HCOV-NL63, HCoV-229E, SARS-CoV, and MERS-CoV [33] and later evolved to transmission hosts such as cattle for HCoV-OC43, alpacas for HCoV-229E, masked palm civets for SARS-CoV and dromedary camels for MERS-CoV [32,34,35]. Bats could be the natural reservoir hosts for several viruses due to their sustained flight, high body temperature, and the tolerance of their immune system against invading viruses for long periods of time [32,36]. In addition, it is believed that the SARS-CoV-2 infection started at a local seafood market in Wuhan, Huanan, that sells fresh bat meat, pangolins, raccoon dogs, and other animals. Worobey M 2021 dissected the early COVID-19 cases in Wuhan, and these findings supported the live-animal market origin of the pandemic [37]. However, the genomic and evolutionary analysis of the SARS-CoV-2 genome showed that SARS-CoV-2 has 96.2% genome similarity to horseshoe bats, *Rhinolophus affinis* CoV RaTG13 and amino acid insertion site similarity to *R. malaynus* RmYN02 between the S1-S2 cleavages. In addition, other phylogenetic investigations have shown that SARS-CoV-2 is closely related to bat CoVs such as ZC45, ZXC21, BANAL-52, BANAL-53, BANAL-103, BANAL-236, RpYN06, PrC31 implicating that SARS-CoV-2 and other SARS-related viruses have likely diverged from common ancestral bat-originated CoVs, diversion may have occurred 40–70 years ago [15,38,39] While the exact origin of SARS-CoV-2 is still unknown, there are recent incidents of SARS-CoV-2-related virus genomes that emerged from bats in different countries [40,41,42,43]. Lytras et al. strongly suggest that bats are likely to be the reservoir species in the emergence of SARS-CoV-2 from an ancestor CoV [44]. However, bats lack direct contact with humans and are less likely to be directly infected with bat coronavirus to provide evidence of bat origin of SARS-CoV-2.

### 4.2. Pangolins

In addition to bat CoVs, pangolins CoVs, especially Malayan pangolins CoV, are believed to be the second closest relative of SARS-CoV-2 after bat CoVs, sharing 85.5% to 92% sequence similarity and 97% amino acid resemblances with SARS-CoV-2 suggesting that pangolins may serve as a potential reservoir or intermediate host for SARS-CoV-2 [14,38,45]. The evolutionary relatedness was implicated from the spike protein analysis of SARS-CoV-2, which suggest that the receptor binding domain of SARS-CoV-2 spike protein is a result of a series of recombination events between the bat CoV RaTG13 and the pangolin CoV MP789 maintained by natural selection and gene drift. Consequently, the SARS-CoV-2 ancestor crossed species to infect humans where the RBD of the S protein has been highly conserved or similar to pangolins-CoV compared to bat-CoV. Moreover, pangolin CoVs (GX/P2V/2017 and GD/1/2019) and SARS-CoV-2 have consistency in their interaction with human ACE2, with a broader host range and binding affinity of pangolin-CoV compared to SARS-CoV-2 [14,45,46]. Several studies showed that the SARS-CoV-2 in pangolins could contribute to SARS-CoV-2 evolution [40,47]. However, more studies are needed to prove the greater relatedness of SARS-CoV-2 to pangolin-derived CoVs and the likelihood of cross-species infection dynamics since some of the studies have suggested they were not the original source of SARS-CoV-2 outbreak [48]. Nonetheless, pangolins naturally carry *Sarbecoviruses*, a subgenus that includes SAR-CoV and SAR-CoV-2 [49,50], which can be a potential cause of future other human coronavirus pandemics [48].

## 5. Natural Infection in Companion and Farm Animals

### 5.1. Domestic Cats and Ferrets

There are several reports in the literature that showed sporadic infection of SARS-CoV-2 in domesticated cats *(Felis catus)* under experimental and natural living conditions globally [51,52,53,54]. The first case of positive SARS-CoV-2 infection in a cat was reported in Brussels [55]. Likewise, other countries have reported SARS-CoV-2 infection in a cat in quarantined COVID-19 households or close contacts based on RT-polymerase chain reaction (PCR). The US Department of Agriculture (USDA) has recorded a total of 118 confirmed cases of SARS-CoV-2 infection in cats via PCR and antibody response studies as of 1 May 2023 [31]. A serological survey of SARS-CoV-2 in cats detected high seroprevalence of antibodies against SARS-CoV-2 ranging from 21–53% in pets (dogs and cats) in COVID-19-positive households [56,57]. Moreover, the SARS-CoV-2 Alpha variant (B.1.1.7), the Lambda variant (C.37), the Delta variant (B.1.617.2), the Epsilon variant (B.1.427/B.1.429) and the Iota variant (B.1.526) were detected in pet cats after exposure to infected owners and from a fully vaccinated but infected human in many countries [31,52,58,59]. Multiple studies have investigated the replication and transmission of SARS-CoV-2 (B.1.1.7) and observed air borne transmission of the virus from animal-to-animal and neutralizing antibodies against SARS-CoV-2 in companion animals [54,60]. Similar to cats, ferrets are also found to be susceptible to SARS-CoV-2 in natural and experimental settings, with virus titer in the upper respiratory track, virus shedding, and active virus transmission [31,54,61]. For instance, the ferret was infected with the SARS-CoV-2 beta variant (B.1.351) to establish the inefficient route for SARS-CoV-2 infection in the ferret model [62]. The Delta variant exhibited an enhanced fitness in vivo ferret model [63]. Compared to cat ACE2, ferret ACE2 shares lower amino acid sequence similarity and mutations in key S-protein binding residues with human ACE2 (hACE2) [54]. In summary, ferrets and cats are susceptible to SARS-CoV-2 infection in natural and experimental conditions; however, spill back reports from these animals to humans are limited. For instance, the suspected cat-to-human transmission of SARS-CoV-2 was implicated from the infected owner to the cat and then from the cat to the veterinarian after the cat sneezed during an examination [64].

### 5.2. Dogs

Dogs (*Canis lupus familiaris*) are broadly reported to be susceptible to SARS-CoV-2 infection and may serve as a transmission host to humans, particularly based on the similar frequency of reduced CG dinucleotides in the SARS-CoV-2 and canine pantropic alpha coronavirus (CCoV) [65,66,67]. Canine ACE2 differs at five positions in the SARS-CoV-2 S RBD and ACE2 interaction and has lower binding ability compared to hACE2 [23]. Sit and Brackman et al. first reported that 2 out of 15 dogs from COVID-19 positive households in Hong Kong, China tested positive for SAR-CoV-2 and showed a reduction in neutralization antibodies during the antibody response study, suggestive of human-to-canine transmission of SAR-CoV-2 [68]. Similarly, several countries have reported confirmed cases of positive SARS-CoV-2 infection in pet dogs living in a COVID-19 positive households [69,70]. The USDA has recorded a total of 115 confirmed cases of different SARS-CoV-2 variants (B.1.1.7, AY.3, AY.43) in dogs through PCR and antibody response studies as of 1 May 2023 [31,52,58,71]. In an experimental study by Shi et al., dogs experimentally infected with SARS-CoV-2 developed a mild infection and shed low viral RNA load in the nasal, oral and rectal swab when compared to high viral load in cases of experimentally infected cats and ferrets, implicating that dogs are susceptible to SARS-CoV-2 infection but may be less than cats and ferrets [54].

### 5.3. Minks

Farmed minks (*Neovison* sp. and *Mustela* sp.) have been reported to be susceptible to SARS-CoV-2, and reports provide evidence of bidirectional animal-to-animal and animal-to-human transmission of the virus in infected farmed minks in several countries [31,72,73,74]. The initial infection in minks started from the infected humans, and the virus has rapidly spread in mink farms with factors associated with mink age, farm distance, size, and infected workers [75,76]. The USDA has reported 18 mink farms with positive COVID-19 cases within the USA as of 1 May 2023 [31]. The mink SARS-CoV-2 genome and mink ACE2 protein analysis detected about 170 mutations in the virus genome and ACE2 polymorphism, which could confer host entry, binding affinity, immune escape, or adaptation of SARS-CoV-2 in minks [77]. The acquired Y453F mutation in the S protein of the mink SARS-CoV-2 showed a reduction in antibody-mediated neutralization [78]. In countries such as the Netherlands and Denmark, the rapid and continued spread of the SARS-CoV-2 in minks had prompted an earlier ban on mink farming [79]. Due to mink infection, the SARS-CoV-2 variant known as Cluster 5, with mutations such as Y453F, 69–70delHV, 1692V, M1229I, and S1147L, was detected in the human population in Denmark [74]. Hence, several SARS-CoV-2 variants in minks were found with mutations such as Y453F, deletion of ORF8, N501T mutation, and others. Mink-to-animal transmission of SARS-CoV-2 was suspected in free-roaming cats through their activity on infected mink farms, and hence a potential risk of transmission extended to household pets, peri-domestic animals, and into neighborhoods, with a potential to spill back to humans [79,80].

To summarize, the public health risk and transmission rate of infection from infected animals back to humans has been considered low; however, it is imperative to investigate multiple hosts and susceptible environments for SARS-CoV-2 spread with the possibility of establishment of natural infection or adaptation in animals prior to spilling back into humans. Furthermore, human-to-animal transmission followed by animal-to-animal circulation and animal-to-human transmission of the SARS-CoV-2 associated with genetic evolution should be discussed thoroughly [73,81]. For instance, the probable hamster-to-human transmission of SARS-CoV-2 variants, AY.127 and B.1.258, has been demonstrated in pet shop-related COVID-19 outbreaks [82,83]. Likewise, the most recent variant of concern (VOC), Omicron (B.1.1.529), may have evolved in mice before spilling to humans based on in silico investigation of mutations in Omicron [84]. Hence, the monitoring of SARS-CoV-2 transmission to or by animals is essential to understand how viruses are evolving in animals, whether new variants are arising from animals, and if new variants pose any risk to public health.

## 6. SARS-CoV-2 Variants

**Figure 4:**
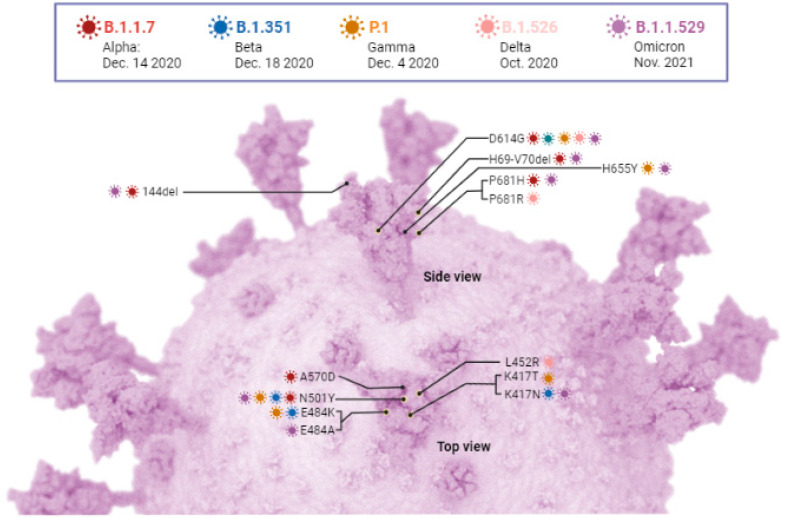
**The key amino acid mutations on the S proteins of five variants of SARS-CoV-2.** Mutations occur on the S protein subunits and other areas of the SARS-CoV-2 genome, giving rise to different VBMs and VOCs. The key mutations on the S protein of SARS-CoV-2 are marked with black lines at the indicated positions in different variants (figure created with Biorender.com and accessed on 10 May 2023).

Several variants of SARS-CoV-2 have been evolving, dominating, or waning globally for the last three years. Accumulation of amino acid mutations and recombination events in the genome has led to the continuous emergence of SARS-CoV-2 variants with altered transmissibility, virulence, effectiveness of treatment or vaccines, and neutralizing capacity (Figure 4). Some major variants being monitored (VBMs) and VOCs surpassing the local or national spread are discussed below and summarized in Table 2 and Table 3.

### 6.1. SAR-CoV-2 Variant Being Monitored and Variant of Concern

#### 6.1.1. Alpha Variant (B.1.1.7)

The Alpha variant of pango lineage B.1.1.7 was the first VOC reported by the WHO in September 2020 in Southeastern England, UK, and has rapidly spread worldwide [85,86]. The Alpha VOC was later designated as a VBM in September 2021. All other initially designated VOCs (Alpha, Beta, Gamma, and Delta variants) have been designated VBMs after 21 September 2021 [86]. The alpha variant was the most prevalent SARS-CoV-2 variant in the US and Europe during the second wave of COVID-19 [87,88]. Several epidemiological reports in various countries have shown that the Alpha variant has an increase of around 40–80% transmissibility, a higher rate of hospitalization, and (12–64%) increased risk of death when compared to the original SARS-CoV-2 strain [85,89,90,91,92,93,94]. For instance, Davies et al. showed that out of 17,452 COVID-19 deaths, nearly 5000 deaths were due to this variant in the UK and estimated an increase of 55% mortality due to the Alpha variant compared to other circulating variants [91,92]. B.1.1.7 has accumulated about twenty-three mutations in its genome compared to the original Wuhan strain, of which eight mutations are found to be in the RBD of the S protein. The most significant mutations in the Alpha variant include deletions of 69–70 and Y144, N501Y, A570D, D614G, P681H, T716I, and S982A. The N501Y and H69/V70 mutations on S protein have been shown to contribute towards more contagious behavior and enhanced affinity for the ACE2 receptor and hence, the enhanced infectivity, while the P681H mutation, located adjacent to the furin cleavage site, may affect cell entry and transmissibility [95,96,97,98]. There were additional concerns when the Alpha variant bearing the E484K with reduced neutralization ability (50%) by therapeutic antibodies and enhanced immune escape capacity, attributed to deletions of 69–70 and 144, was identified in the UK and Europe [87,99,100]. Moderna and Pfizer have assured that their vaccine would be effective against B.1.17 as both mRNA vaccines target the S protein, where the most notable mutations occurred. A study by Abu-Raddad and Chemaitelly estimated 89.5% effectiveness of Pfizer-BioNtech BNT162b2 against B.1.1.7 variant in a cohort study carried out in Qatar while the vaccine is nearly 100% effective in preventing severe cases or fatality in case of the original Wuhan strain [101]. In addition to these vaccines, NVX-CoV2373, a protein subunit vaccine made by Novovax company, is shown to be 85.6% effective against this variant as compared to 95.6% effectiveness against the original variant of SARS-CoV-2 [102]. Several studies have concluded that variant B.1.1.7 has a moderate reduction in neutralization by therapeutic antibodies from the serum of convalescent individuals (1.5–10-fold) and several COVID-19 vaccine recipients (~2-fold) [99,103,104]. Re-infection and reoccurrence are less frequent with the Alpha variant compared to other variants as this variant is susceptible to available anti-COVID-19 vaccines [105]. Before the Delta variant became prominent, the Alpha variant comprised 66% of cases in mid-April 2021, according to a CDC study released in June 2021 [106].

#### 6.1.2. Beta (B.1.351)

The Beta variant, lineage B.1.351, was first reported in October 2020 in Nelson Mandela Bay in South Africa and spread worldwide, including Zambia in December 2020 and the US in April 2021 [107]. The Beta variant has an estimated 50% increased rate of transmission, with community transmission mostly occurring in North America, Europe, and Africa [108]. The Beta variant harbors about twenty-three new mutations compared to the original strain, among which nine mutations were found on the S protein RBD region. The most notable mutations in the Beta variant include K417N, E484K, and A701V, along with N501Y and D614G, the latter two are similar to the Alpha variant [107]. Numerous studies reported that E484K substitution alone or in combination with N501Y and K417N were associated with a reduction in neutralization as well as an increase in ACE binding affinity and transmissibility of the Beta variant [109,110]. Several other studies have also supported a significant level of decreasing neutralizing antibodies against B.1.351 infection with convalescent plasma (11–33 fold) and post-vaccination antibodies (3.4–8.5 fold), making it more susceptible to host immune evasion and resistance to antiviral treatment [99,111,112,113,114,115]. In addition, re-infection is possible, and vaccine effectiveness is lowered [116,117,118,119]. Pfizer-BioNtech has reported that its vaccine would be about 72% effective against the Beta variant [101,116,117,118,119]. Moreover, NVX-CoV2373 was reported to be 60% protection against this variant compared to 95.6% effectiveness against the original variant of SARS-CoV-2 [102]. Abu-Raddad and Chemaitelly recorded breakthrough infections (0.66 per 1000 people) in people who had received one dose and both doses of the vaccine [101]. Delphine et al. demonstrated that B1.351, but not B.1.1.7, may increase the risk of infection in immunized individuals [114].

**Table 2:** Summary of the SARS-CoV-2 variant and current status (VBM and VOC), lineages and descendent lineages, earliest documentation in country and date, key mutations, phenotypes transmissibility, and neutralization activity.

Variants and Current Status	Lineages and Descendent Lineages	Country and Date of Earliest Identification/Documentation)	Key Mutations Reported	Phenotypes	References
Alpha VBM	B.1.1.7 and Q lineages	UK, September 2020	69/70 del **, Y144 del **, N501Y **, A570D, D614G **, P681H **, T716I, S982A, D1118H	Increased transmissibility (30–80%); increased risk of hospitalization (12–64%) and risk of death (30–70%); no adverse effects on vaccine efficacy	[85,86,87,90,92,95,96,97,98,101,104,115]
Beta VBM	B.1.351 and descendant lineages	South Africa, October 2020	L18F **, D80A, D215G, 241–243 del, K417N **, E484K **, N501Y **, D614G **, A701V	Increased transmissibility; increased risk of death during hospitalization; immune escaping capacity	[101,107,109,110,111,112,113,114,119]
Gamma VBM	P.1 and descendent lineages	Brazil, May 2020	L18F **, T20N, P26S, D138Y, R190S, K417T **, E484K **, N501Y **, D614G **, H655Y **, T1027I, V1176F	Increased transmissibility; less severe; immune escaping capacity	[86,90,120,121,122,123]
Delta and Delta Plus VBM	B.1.617.2 And descendent lineages	India, October 2020	T19R, E156G, 157–158 del, K417N ^++^, L452R, T478K, D614G **, P681R, D950N	Increased transmissibility; less severe; immune escaping capacity	[86,89,115,124,125,126,127,128]
Omicron VOC	B.1.1.529 and descendent lineages (BA.2.75, BQ.1, BQ.1.1, BF.2.3.20, BF.7, BF.11, XBB, XBB.1.5, XBC, XAC BN.1, CH.1.1)	South Africa, November 2021	D3G, P13L, Q19E, 31–33 del, A63T, A67V, 69–70 del **, T91, T95I, 142–144 del **, R203K, G204R, del211, L212I, ins214EPE, G339D, S371L, S373P, S375F, K417N **, N440K, G446S, L452R ^++^, S477N, T478K, E484A, Q493R, G496S, Q498R, N501Y **, Y505H, T547K, D614G **, H655Y **, N679K, P681H **, N764K, D796Y, N856K, Q954H, N969K, L981F	Highly increased transmissibility, less severe disease development (>20%); increased impact on immunity and re-infection, potential reduction in neutralization	[43,86,129,130,131,132,133,134,135]

** indicates mutation that appears in two or more variants. ^++^ indicates mutation that appears in their descendants but not in the original lineage.

#### 6.1.3. Gamma (P.1)

The Gamma variant P.1, lineage B.1.1.28.1, first appeared in Manaus, Brazil, causing a widespread infection in May 2020 and was later reported by Japan’s National Institute of Infectious Disease (NIID) in January 2021 [120]. The Gamma variant carries 12 mutations in the S protein, with the most notable mutations being L18F, T20N, P26S, D138Y, R190S, H655Y, and T1027I, along with the previously reported K417T, E484K, N501Y, and D614G mutations [86,89,120,121,136]. The combined effect of these mutations has caused a significant increase in ACE binding affinity, immune evasion, transmissibility, and breakthrough infections that led to increased cases of hospitalization due to the Gamma variant compared to other previous variants [90,122,137]. A study by Navaca et al. suggested that the P.1 variant could have a nearly 2.2% higher rate of transmission than its parental lineage B.1.1.28, which may lead to fewer re-infection cases of the P.1 variant with COVID-19. However, adults (18–59 years old) infected with the P.1 variant are around 10% more infectious than those infected with non-P.1 SARS-CoV-2 viruses [122]. There were several reports of the resurgence of circulating variants (B.1.1.7, P.1, P.2 (Zeta), and P.3 (Theta)) around the end of 2020 in several locations in Europe, including Brazil, which had become the COVID-19 epicenter during the first wave of COVID-19 pandemic [120,122,138,139]. Several articles have shown that the neutralization ability of the P.1 variant ranges from 6.5 to 13-fold and 2.2 to 2.8-fold for convalescent plasma and post-vaccination serum, implicating that neutralization ability against P.1 variants is slightly weakened compared to Alpha B.1.1.7; however, it is not as substantial as that of Beta B.1.351. Similar neutralizing behavior of the Gamma variant and Beta variant could be due to a similar triple mutation in RBD of the S protein of SARS-CoV-2 in both variants [103,123,136]. The vaccine effectiveness of full vaccination against the Gamma variant was estimated at about 72–82%, evaluated by Shao et al. 2022 [140]. The same study evaluated the vaccine effectiveness of full vaccination against the Alpha, Beta, and Omicron variants ranging from 86.8–96% for Alpha, 70.9–72.8% for Beta, 84.9–90.3% for Delta, and 56.5–82.4% for Omicron variant of SARS-CoV-2 [140].

#### 6.1.4. Delta (B.1.617.2)

The Delta variant, lineage B.1.617.2, was first reported in Maharashtra, India, in October 2020 and spread to the UK in March 2021 and to the US in April 2021 [86]. According to the WHO, the Delta variant and Delta plus variants (AY lineages) are the fastest and fittest variants found to be more contagious, more infectious, and have higher immune evasion ability [127,128]. The Delta genome has about 10 more mutations in the spike protein RBD than the original SARS-CoV-2 strain, with the most notable mutations being D111D, G142D, del 156–157, R158G, L452R, T478K, D614G, P681R, and D950N [141,142]. The Delta variants have acquired additional mutations (K417N substitution) in the S protein leading to the so-called “Delta Plus” variants (AY.1, AY.2, and AY.4.2 sub-lineages) that had an increased infection rate of 60% compared to the Delta variant because of their higher affinity for lung cells [143]. Collectively, these mutations in the S protein could confer higher transmissibility (D614G and P681R), higher infectivity (L452R, K417N, and T478K), or evading neutralizing antibodies formed after infection or vaccination, i.e., greater immune escape ability (T478K) [124,125,126]. The Covaxin vaccine from Bharat Biotech has reported about 78% effectiveness against this variant [144]. The Delta variant is about twice more contagious than the original strain and 60% more infectious than the Alpha B.1.1.7 variant [126]. Other studies on the Delta variant support that the Delta variant could cause severe diseases and have higher case fatality rates in unvaccinated patients compared to patients infected with the Alpha variant, Omicron variant, or the original Wuhan strain [145,146,147,148]. Researchers also found an increase in the relative odds of long COVID with the Delta variant depending on age and time since vaccination compared to Omicron variants [149]. The Delta variant has demonstrated a decrease in the effectiveness of COVID-19 vaccines (96% for Oxford-AstraZeneca, 92% for Pfizer-BioNtech) against SARS-CoV-2 infection and hospitalization patients compared to previous variants [150]. Likewise, the Delta variant has a reduction in neutralization immunity by convalescent sera and post-vaccination sera [115,124,151,152].

#### 6.1.5. Omicron (B.1.1.529)

The most recently designated VOC, Omicron, was first detected in Botswana and South Africa in November 2021 and has spread to most countries [86]. The WHO has warned the global public about the enhanced immune escape potential, higher transmissibility, and the global spread of Omicron, particularly in countries with low vaccination rates [86,130]. The Omicron variant harbors about 60 unique mutations overall, with 15 S protein mutations and other nonsynonymous mutations in the S protein. The most notable mutations include T478K, Q493R, Q498R, Y505H, and H655Y, along with the previously reported 69–70 del, 142–144 del, K417N, E484A, D614G, N501Y, and P681H mutations and the combined effect of these mutations has been reported to contribute toward greater ACE2 binding affinity, increased transmissibility, enhanced immune evasion ability, increased disease risk and other modified behaviors of Omicron compared to previously reported variants [132,133]. The Omicron variant exhibits greater escape activity and very low neutralization activity from therapeutic antibodies compared to previous variants [153]. Several studies have shown that Omicron infections cause milder health impact than other variants, for instance, a 41% hospitalization rate, 18–20% severe disease development, and 3% mortality as compared to a 69% hospitalization rate, 30–74% severe disease development, and 29% mortality in the case of the Delta variant [153,154]. However, the acquired mutations in the Omicron variant lead to five main Omicron lineages, including BA.1, BA.2, BA.3, BA.4, and BA.5, which spread faster than the previous Omicron (B.1.1.529). BA.4/5 was found to be more transmissible and infectious than other Omicron lineages, and this behavior could attribute to the presence of L452R mutation in these lineages but not in all Omicron lineages [134,155]. Recently, several sub-lineages of the Omicron, such as BA.2.75, BQ.1, BQ.1.1, BF.2.3.20, BF.7, BF.11, XBB, XBB.1.5, XBC, XAC BN.1, CH.1.1 and others have also evolved. The Omicron sub-lineages are the dominant SARS-CoV-2 variants in the USA in 2023, and they showed immune evasion capacities and effectiveness against some antiviral drugs [43,129,130,131]. For the Omicron variant, the infection prevalence was 90% as compared to 80% for Delta and 50% for the Beta variant [134]. Co-infection of the Omicron or Delta variant is also reported in immunocompromised hosts [156]. A study in Norwegian households showed that the secondary attack rate of SARS-CoV-2 was higher with Omicron variants than with the Delta variant [157]. The COVID-19 vaccines such as Pfizer-BioNTech, Moderna, AstraZeneca, Covaxin, and Covidshield have about 95%, 94.1%, 76%, 65.2%, and 90% efficacy against the Omicron variants, respectively, in India [134]. In addition, studies have shown that booster immunization with available vaccines has increased vaccine efficacy and helped to prevent the spread of the Omicron variant and hospital admission compared to two doses of vaccines [158,159,160,161]. A case-control study from Qatar observed 90% re-infection protection against the Alpha variant, 86% against the Beta variant, and 92% against the Delta variant, but only 56% and 88% protection against the Omicron and severe Omicron diseases, respectively [162]. In addition, the fourth dose of mRNA vaccination provided adjusted protection rates of 34%, 67%, and 72% against overall infection, hospitalization, and related death during the Omicron rise [135].

#### 6.1.6. Mu (B.1.621)

The Mu variant, a sub-lineage B.1.621.1, was first detected in Colombia in January 2021 and was designated as a VOI in August 2021 and VBM in September 2021, as this variant has caused sporadic cases globally [86,163]. This variant has caused larger outbreaks in some countries, such as Colombia and Ecuador, and has spread to other countries, including Mexico, Spain, Japan, and Hong Kong [163,164]. Preliminary reports have implicated that the Mu variants may have higher host cell entry, therapeutic resistance, and partial immune evasion of preexisting immunity [165,166,167]. The Mu variants accumulate a 21 new mutations with nine mutations in the S protein, and significant S protein mutations include T95I, Y145S, 146N insertion, R346K, E484K, N501Y, P681H, and D950N. The R346K and E484K mutations may confer the immune escape property in the Mu variant, as this mutation has been shown to reduce neutralizing antibodies by convalescent and post-vaccinated sera in previous variants [168,169]. Uriu et al. have demonstrated that the Mu variant is highly resistant to the antibodies elicited from convalescent individuals (12.4-fold) and Pfizer-BioNtech vaccinated individuals (7.6-fold) when compared with the Beta variant (B.1.351) [165].

#### 6.1.7. Lambda (C.37)

The Lambda variant, also known as lineage C.37, was first reported in December 2020 in Peru and designated as a VOI by the WHO in June 2021 and VBM in September 2021 [86]. This variant became the predominant strain in Peru in March 2021 (81.82%) and has spread to the rest of the world, mostly in South America [170]. The most significant mutations in the Lambda variant are the 246–253delinsN, L452Q, F490S, and D614G in the S protein RBD [141]. The L452Q mutation alone enhances ACE2 binding affinity and infectivity, while combined with F490S and 246–253delinsN, it contributes towards neutralization immunity and vaccine escaping capacity post-vaccination [100,171,172,173,174]. The pseudo-typed virus with Lambda S is found to have higher transmissibility or rendered neutralizing antibodies (2.3–3.3-fold) by convalescent sera and post-vaccination antibodies, particularly with the adenoviral vector-based COVID-19 vaccine (Ad26.COV2.S) compared to mRNA vaccines (BNT162b2 and mRNA-1273) [170,171,174,175]. Carreno et al. reported a reduction in the neutralization activity using mRNA vaccines against Lambda C.37 (4.6-fold), Beta B.1.351 (4.2-fold), the Alpha B.1.1.7 plus E484K (3.8-fold) and the Delta B.1.617.2 (3.0-fold) variants isolated from clinical specimens compared to the wild-type [100].

#### 6.1.8. Epsilon (B.1.427/B.1.429)

The Epsilon variant, including two separate lineages, B.1.427 and B.1.429, was first identified in California, US, and reported as a VOC by CDC in September 2020. However, the WHO has entitled this variant as a VOI, not as a VOC, in March 2020 and a VBM in September 2021. The lineage B.1.427, which harbors two notable S mutations, L452R, and D614G, was first identified in May 2020 but has spread rapidly between September 2020–January 2021 in the US. The latter lineage B.1.429, harbors four mutations, S13I, W152C, L452R, and D614G, in the RBD of the S protein [86,176,177,178,179]. The S131 and W152C S protein mutation increases the infectivity of the variant, while L452R promotes the binding affinity of the S protein for ACE2 and viral replication capacity [177,178,180]. Studies have shown that Epsilon variants have a moderate reduction in neutralization via convalescent serum and post-vaccination recipient serum by 4–6.7-fold and 2–2.9-fold, respectively [177,181]. Multiple studies reported the SARS-CoV-2 variants B.1.427/B.1.429 could escape the host immune system and cause severe illness [177,182].

#### 6.1.9. Kappa (B.1.617.1)

The Kappa variant, B.1.617.1, is a sub-lineage of B.1.617 (Delta variant) with S protein RBD mutations such as L452R and E484Q. It was first reported in India in December 2020 and designated as a VOI in March 2021 and a VBM in September 2021 [86]. B.1.617.1 harbors important mutations in the S protein, such as T19R, G142D, E154K, D614G, and P681R [179]. Collectively, these mutations, particularly E484Q, L5452R, and T478K, may affect the transmissibility, binding affinity of S protein to ACE2 (affinity lies between the original and the Gamma variant), immune escape ability and reduction in neutralization activity [115,151,183,184].

Some of these VBMs that have evolved or circulated worldwide are summarized in Table 3.

**Table 3:** Summary Table of the lineage, earliest documentation, and key mutations in the SARS-CoV-2 VBMs.

SARS-CoV-2 Variants	Current Status Designation	Lineage	Earliest Identification/ Documentation (Country, Date)	Key Mutations	References
Mu	VBM	B.1.621 and B.1.621.1	Colombia, January 2021	T95I, Y144S, Y145N, R346K, E484K, N501Y, D614G, P681H, D950N	[86,163,166,169]
Lambda	VBM	C.37 B.1.1.1.C37	Peru, December 2020	G75V, T76I, 246–253 delinsN, L452Q, F490S, D614G, T859N	[86,100,174,179]
Kappa	VBM	B.1.617.1	India, October 2021	T95I, G142D, E154K, L452R, E484Q, D614G, P681R, Q1071H	[86,115,179,183,184]
Epsilon	VBM	B.1.427 and B.1.429	USA, September 2020	B.1.427: L452R, D614G B.1.429: S13I, W152C, L452R, D614G	[86,177,178,179,180]
Eta	VBM	B.1.525	Nigeria, UK, December 2020	Q52R, A67V, 69/70 del, 144 del, E484K, D614G, Q677H, F888L	[86,185,186]
Iota	VBM	B.1.526	USA, December 2020	L5F, T95I, D253G, S477N, E484K, D614G, A701V	[86,94,179]
Theta	VBM	P.3	Philippines January 2021	Del 141–143, E484K, N501Y, D614G, P681H	[86,187,188]
Zeta	VBM	P.2/B.1.1.28.2	Brazil and Japan	L18F, T20N, P26S, A119S, F120F, F157L, M234I, E484K, D614G, S929I, V1176F, L3468V	[86,179,181,189]

## 7. Conclusions

The emergence of SARS-CoV-2 variants and their rapid spread has posed a great need for research into viruses, their diseases, and infections in animals and humans. SARS-CoV-2 is the third highly pathogenic human CoV to emerge in the 21st century, which has crossed species barriers to infect humans and animals such as companion, farm, zoo, or wild animals. Correspondingly, new variants of SARS-CoV-2 are continuously evolving, and monitoring each variant is essential to control the rapid spread, even more so when SARS-CoV-2 infection can affect multiple human and animal hosts. The advent of effective therapeutics and vaccines provides hope for controlling the ongoing COVID-19 pandemic; however, the susceptibility of the virus to a broad range of hosts and the continuous evolution of SARS-CoV-2 variants is very daunting. Hence, to fully eradicate COVID-19 in the future, an extensive understanding and surveillance of SARS-CoV-2 epidemiology, reservoir species, animal host susceptibility, variants, and vaccine efficacy is needed, along with effective vaccination and booster immunization to humans and animals.

## Figures and Tables

**Table 1:** Brief description of various structural, non-structural, and accessory proteins of SARS-CoV-2 (Genomic organization of SARS-CoV-2 is shown in Figure 1B).

Gene/Proteins	Function	Gene/Proteins	Function	
ORF-1a and ORF-1b	Encodes replicase polyprotein 1a (pp1a) and polyprotein 1b (pp1b), pp1a (nsp 1–11), and pp1b (nsp 11–16) cleaved into nsps 1–16 (Figure 1B)	Nsp-14	3′-5′ exoribonuclease; RNA cap formation; N-7-guanine methyltransferase	**References for SARS-CoV-2 genes or proteins** **[17,18,19,20,21,22]**
Nsp-1	Restricts host innate immune response; Host mRNA degradation, translation inhibition	Nsp-15	Endoribonuclease; chymotrypsin-like proteinase; evasion of immune response	
Nsp-2	Binds to prohibitin (PHB)-1 and PHB-2; modulates host survival signaling pathway	Nsp-16	2′O-ribose methyltransferase; RNA cap formation; restricts host innate response	
Nsp-3	Interacts with N-protein for double-membrane vesicle (DMV) formation; Papain-like proteinase; cleaves viral polyprotein and assists in immune escape	Spike protein	Forms spike complexes on the virion surface; attaches to host receptor ACE2 for virus entry and subsequent infection	
Nsp-4, 6	Viral replication-transcription transmembrane scaffold protein; assembles DMV	ORF-3a and 3d	Forms ion channels for viral exit; induces apoptosis and pathogenesis	
Nsp-5	M^pro^/3C-like proteinase; cleave viral polyprotein at C-terminus; Inhibition of interferon signaling	Envelope protein	Forms the viral envelop; interferes with host immune responses (apoptosis)	
Nsp-7	Complexes with nsp-8 and nsp-12; Cofactor for RNA-dependent RNA polymerase	Membrane protein	Most abundant; bind to the N for virus morphogenesis and assembly; transmembrane transport of nutrients and bud release	
Nsp-8	Makes heterodimer with nsp-7 and nsp-12; primase	ORF-6	Membrane-associated protein in the ER and Golgi compartments	
Nsp-9	Complexes with Nsp-8; binding of single-stranded RNA; protein phosphatase	ORF-7a and 7b	Transmembrane protein; involved in viral escape	
Nsp-10	Cofactor for nsp-14 and nsp-16 to cap viral RNA	ORF-8	Role in host immune response	
Nsp-12	RNA-dependent RNA polymerase/replicase; nucleotidyltransferase	Nucleocapsid protein	Warps the viral RNA; facilitates M proteins during virus assembly; Restricts antiviral response	
Nsp-13	Helicase; RNA 5′ triphosphatase	ORF-10	Role in antiviral immune response	

## Data Availability

Data in this review article can be used or presented for research and analysis purposes by appropriately citing the article. Any inquiry can be made with either the corresponding author at her email: qmatthews@alasu.edu or the first author at her email: rpandit6015@myasu.alasu.edu.
